# NOS2-derived low levels of NO drive psoriasis pathogenesis

**DOI:** 10.1038/s41419-024-06842-z

**Published:** 2024-06-26

**Authors:** Ines Köhler, Cecilia Bivik Eding, Nada-Katarina Kasic, Deepti Verma, Charlotta Enerbäck

**Affiliations:** Ingrid Asp Psoriasis Research Center, Department of Biomedical and Clinical Sciences, Linköping University, Linköping, Sweden

**Keywords:** Inflammation, Psoriasis

## Abstract

Psoriasis is an IL-23/Th17-mediated skin disorder with a strong genetic predisposition. The impact of its susceptibility gene nitric oxide synthase 2 (NOS2) remains unknown. Here, we demonstrate strong NOS2 mRNA expression in psoriatic epidermis, an effect that is IL-17 dependent. However, its complete translation to protein is prevented by the IL-17-induced miR-31 implying marginally upregulated NO levels in psoriatic skin. We demonstrate that lower levels of NO, as opposed to higher levels, increase keratinocyte proliferation and mediate IL-17 downstream effects. We hypothesized that the psoriatic phenotype may be alleviated by either eliminating or increasing cellular NO levels. In fact, using the imiquimod psoriasis mouse model, we found a profound impact on the psoriatic inflammation in both IMQ-treated NOS2 KO mice and wild-type mice treated with IMQ and the NO-releasing berdazimer gel. In conclusion, we demonstrate that IL-17 induces NOS2 and fine-tunes its translation towards a window of proinflammatory and hyperproliferative effects and identify NO donor therapy as a new treatment modality for psoriasis.

## Introduction

Psoriasis is a chronic, immune-mediated inflammatory skin disease with strong genetic susceptibility [1]. It is a painful and disabling condition, which affects 2–3% of the population in the Western world. The distinctive skin eruptions are characterized by intense hyper-proliferation and disturbed maturation of the epidermal cells and an inflammatory dermal infiltrate. Genome-wide association studies (GWAS) support important roles of the innate immunity, IL-23/Th-17 immune axis and NFkB signaling in the pathogenesis of psoriasis [2].

Nitric oxide synthase 2 (NOS2) has been identified as a susceptibility gene in psoriasis, as well as in other inflammatory disorders [3, 4]. A functional role of NOS2 in psoriasis pathogenesis is further supported by its distinctively upregulated gene expression in psoriatic involved skin in contrast to low expression in different clinical types of eczema. NOS2 expression was consequently suggested as a psoriatic disease classifier [5, 6].

Nitric oxide (NO) is endogenously synthesized from arginine by NO synthases (NOS), of which NOS2 is the inducible isoform and NOS1 (neuronal NOS) and NOS3 (endothelial NOS) are constitutionally expressed at low levels [7]. NO is a pivotal signaling molecule involved in highly diverse biological functions, including the regulation of immune response, apoptosis, vascular dilation, angiogenesis and platelet inhibition [8]. NO thus plays multiple roles in normal physiology and displays aberrant expression in multiple disorders.

In cancer biology, NO has demonstrated anti-tumor as well as tumor-promoting properties, as well as pro- and anti-inflammatory effects [8, 9]. These discrepancies have been attributed to multiple aspects, including the concentration and duration of NO exposure, an oxidative/reductive environment, cell-specific production of NO and tissue localization, all of which have a profound impact on the different outcomes [10]. Consequently, NO/NOS have been proposed as promising targets in the regulation of carcinogenesis and have recently been linked to the effectiveness of cancer immunotherapy [11, 12]. Despite strictly benign cell control, psoriatic cells share several features with cancer, such as disturbed differentiation, hyperproliferation and angiogenesis. Therefore, consequences of NOS2-derived NO, which are observed in cancer tissue, may have similar implications in the microenvironment of psoriatic skin.

The complex interplay between psoriasis susceptibility genes and environmental factors underlying the disease-specific alterations in the skin remains to be fully elucidated. Here, we investigate the functional significance of the susceptibility gene NOS2 in the pathogenesis of psoriasis and translate our findings into a novel therapeutic strategy.

## Materials and methods

### Human skin biopsies

Punch biopsies (4 mm) from psoriatic skin (involved and uninvolved areas) were obtained from patients at the Department of Dermatology at Linköping University Hospital. The patients were examined by a dermatologist and were not receiving any systemic treatment. Following incubation with 3.8% ammonium thiocyanate for 30 min at room temperature, the epidermal layer was separated from the dermal part of the biopsy.

### Cells culture conditions

Primary human epidermal neonatal keratinocytes, HEKn (C0015C, Cascade Biologics, Portland, OR), were cultured in complete EpiLife medium supplemented with 1% EpiLife defined growth supplement (EDGS), CaCl_2_ (0.06 µM; all from Cascade Biologics), 1% amphotericin B (Gibco, Gaithersburg, MD), and 1% penicillin/streptomycin (Gibco).

Cells were treated with IL-17A (7955-IL, 10 ng/ml), IL-22 (782-IL, 20 ng/mL), TNF-α (210-TA, 10 ng/mL), IL-1β (201-LB, 10 ng/ml) or IL-6 (206-IL, 10 ng/mL) separated or in combinations (all from R&D Systems, Minneapolis, MN), for 48 h. For the starvation condition, the EDGS supplement was excluded, and for hypoxia, the keratinocytes were incubated in a 1% O_2_ hypoxia chamber for 48 h. For ROS treatment, the antioxidant inhibitor of GSH synthesis, L-buthionine sulfoximine, BSO (B2515, 100–500 µM, Sigma-Aldrich, St. Louis, MO), was added.

In some experiments, the cells were pretreated for one hour with the NOS2 inhibitor aminoguanidine (AG, 5 mM, #396494, Sigma-Aldrich) before cytokines were added. The NO donor DETA*-*NONOate (#82120, Cayman Chemicals, Ann Arbor, MI*)* was used at concentrations of 0.1–1000 µM.

### RNA extraction, cDNA synthesis and qPCR analysis

Skin biopsies were homogenized using a TissueLyser (Qiagen, Hilden, Germany) and total RNA was extracted with the RNeasy kit for fibrous tissue (Qiagen), according to the manufacturer’s instructions. Cells and epidermis layers were lysed in RLT plus buffer and RNA was isolated using the RNeasy Plus kit (Qiagen).

The cDNA synthesis was performed using the Maxima First Strand cDNA Synthesis Kit (Thermo Fisher Scientific, Waltham, MA) and the gene expression was analyzed by qPCR, using the TaqMan Fast Advanced Master Mix and the following pre-designed TaqMan Gene expression assays; *NOS2* (Hs00167248_m1), *CXCL1* (Hs00236937_m1), *CXCL5* (Hs01099660_g1), *CXCL8* (Hs00174103_m1), *S100A8* (Hs00374264_g1), *BD2* (Hs00175474_m1), *MMP1* (Hs00899658_m1), *MMP12* (Hs00159178_m1), *IL-6* (Hs00174131_m1), *LCN2* (Hs01008571_m1), *PCNA* (Hs00427214_g1), *MKI67* (Hs04260396_g1), *NOS1* (Hs00167223_m1), *NOS3* (Hs01574659_m1) and *RPLP0* (Hs99999902_m1) (Thermo Fisher Scientific). qPCR was performed on a real-time 7500 HT system (Applied Biosystems, Foster City, CA) and samples were run in triplicate. The data were normalized to the housekeeping gene *RPLP0*, using the comparative Ct (2^−∆∆Ct^) method. For mice samples, the pre-designed TaqMan Gene expression assays for *Nos2* (Mm00440502_m1), *Rplp0* (Mm00725448_s1) (Thermo Fisher Scientific) were used.

miRNA isolation was performed from cells with the Allprep DNA/RNA/miRNA Universal kit and the miRCURY LNA RT kit and miRCURY LNA SYBR Green PCR kit (all from Qiagen) were used for cDNA synthesis and qPCR analysis respectively, according to the manufacturer’s instructions. The specific primer assays, hsa-miR-146a-5p (YP00204688) and hsa-miR-31-5p (YP00204236), were used and hsa-miR-26a-5p (YP00206023) was used for normalization (Qiagen) with the comparative Ct (2^−∆∆Ct^) method.

### ELISA

Analyses of MMP1 and CXCL1 protein levels in conditioned medium were performed using the human pro-MMP1 and CXCL1/GROα Quantikine ELISA immunoassay kits (DMP100, DGR00B, R&D Systems, Minneapolis, MN), according to the manufacturer’s instructions. The absorbance was measured in a VersaMax microplate reader (Molecular Devices, San Jose, CA) and the samples were analyzed in duplicate. Protein levels were presented as ng/mL.

### Immunofluorescence

Human skin punch biopsies of psoriasis-involved skin and healthy control skin were fixed in 4% formaldehyde and paraffin-embedded. Sections were cut at 5 μm, deparaffinized in Histolab-clear (Histolab Products, Gothenburg, Sweden) and rehydrated in ethanol. The sections were incubated with citrate antigen retrieval buffer (pH = 6, Invitrogen, Carlsbad, CA) at 97 °C and blocked with 5% bovine serum albumin for 45 min at room temperature. The sections were either stained with hematoxylin and eosin (H&E, Histolab Products) or incubated with the following primary antibodies; iNOS (Novus Biologicals, NB300-605, Littleton, CO; Thermo Fisher Scientific, PA3-030A; Calbiochem, 482728; Abcam, Cambridge, UK, ab3523) or 3-Nitrotyrosine (Abcam, ab61392) overnight at 4 °C and secondary Alexa Fluor 555 conjugated antibody (Molecular Probes, Eugene, OR, USA) for one hour concurrently with DAPI (4′,6-diamidino-2-phenylindole) to counterstain the nuclei. Using the isotype control IgG antibody (NBP2-24891, NBP1-97019, Novus Biologicals) or by omission of the primary antibody, negative controls were achieved and showed no staining. No blinding method was used. Images were examined in a Leica DMi8 inverted fluorescent microscope (Leica Microsystems GmbH, Wetzlar, Germany) and fluorescence intensities were measured using the ImageJ software (National Institutes of Health). For analysis of H&E-stained sections an Olympus BX 51 microscope (Olympus Corporation, Tokyo, Japan) was used.

For mouse skin sections, the same protocol was applied but with the following primary antibodies: CD45 (ab10588, Abcam), Ly6B.2 (NBP2-13077, Novus Biologicals), PCNA (ab29, Abcam) and Ki67 (ab16667, Abcam), and 3-Nitrotyrosine (Abcam, ab61392).

HEKn were fixed in 4% formaldehyde and permeabilized with 0.1% saponin, followed by incubation with an iNOS primary antibody (Novus Biologicals, NB300-605) overnight at 4 °C and subsequent incubation with secondary Alexa Fluor 555 conjugated antibody (Molecular Probes).

### miRNA inhibitor transfection

HEKn were transfected with 10 nM of the miRVana miRNA inhibitors; hsa-miR-146a-5p (MH10722), hsa-miR-31-5p (MH11465) or miRVana miRNA Inhibitor Negative Control #1, using the Lipofectamine RNAiMAX transfection regent (all from Thermo Fisher Scientific) in Opti-MEM (Gibco), following the manufacturer’s protocol. The transfection medium was replaced with Epilife cell culturing media with supplements the day after transfection.

### Nitric oxide detection

The cell-permeable fluorescent probe DAF-FM DA (4-amino-5-methylamino-2′,7′-difluorofluorescein diacetate, D23842, Invitrogen) was used for the detection of intracellular NO. HEKn were incubated with 0.1 µM DAF-FM in culture medium for 15 min at 37 °C prior to the detachment of cells using trypsin. After washing, the cells were directly analyzed in a Cytek Aurora flow cytometry (Cytek Biosciences Inc, Fremont, CA, USA). The measurements and unmixing were performed using the SpectroFlo® Software (Cytek Biosciences Inc.) The cells were unmixed using the reference control and mean fluorescence intensity (MFI) was measured.

### Crystal violet proliferation assay

HEKn were seeded (1500 per well) in a 12-well plate and the culture medium was replaced and new NO donor (DETA-NONOate) was added every third day. After 8 days of culture in the presence of the NO donor, the colonies were fixed in 4% formaldehyde for 10 min at room temperature. The colonies were then stained with 0.05% crystal violet for 20 min, washed with water and air-dried. The stained cells were dissolved in 1% sodium dodecyl sulfate (SDS) solution on a shaker at room temperature for 3 h and the absorbance was measured at 550 nm in a VersaMax microplate reader. Each treatment was carried out in triplicate.

### Protein extraction and western blot analysis

Cells were lysed in RIPA buffer (#89900, ThermoFisher Scientific) supplemented with Halt Protease and phosphatase inhibitor cocktail (#78440, ThermoFisher Scientific). Protein quantification was performed using BCA Protein Assay Kit (#23337, ThermoFisher Scientific), according to the manufacturer’s instructions. Equal amounts of protein (30 µg) mixed with Laemmli sample buffer supplemented with DTT, were denatured for 5 min at 98 °C, separated on a 10% Mini-PROTEAN TGX Precast Protein Gel (#4561034, Bio-Rad Laboratories, Hercules, CA, USA) and transferred to a polyvinylidene fluoride (PVDF) membrane, using the Transfer Blot Turbo PVDF transfer pack (#1704156, Bio-Rad Laboratories) in the Trans Blot Turbo Transfer System (Bio-Rad Laboratories). After blocking in EveryBlot Blocking buffer (#12010020, Bio-Rad Laboratories), the membrane was incubated with primary antibodies against NOS2 (ab3523, Abcam) and β-actin (Invitrogen, #AM4302) at 4 °C overnight and then incubated with corresponding HRP-conjugated secondary antibodies (Amersham Biosciences, Buckinghamshire, UK) for one hour at room temperature. The protein expression bands were visualized with the ECL Plus Western blotting chemiluminescence reagent (#32132, ThermoFisher Scientific) in a ChemDoc Imaging unit (Bio-Rad Laboratories) and quantified by densitometry using Image Lab software (v.6.1.0, Bio-Rad laboratories). NOS2 expression was normalized to β-actin. Uncropped Western blots are presented in the Supplemental material.

### Mouse experiments

Female wild-type (C57BL/6) and iNOS knockout (B6.129P2-NOS2tm1Lau/J) mice (6 weeks) were obtained from Jackson Laboratory (Bar Harbor, ME). The mice were randomly divided into the four groups. To induce a psoriasis-like inflammation, 62.5 mg Aldara cream (5% imiquimod, Meda AB, Solna, Sweden) was applied topically on a shaved back region (3 × 2 cm) for four consecutive days. Control mice were sham-treated with Vaseline cream (ACO, Kista, Sweden). The mice were sacrificed after five days and skin was collected.

Daily topical treatment with 62.5 mg berdazimer sodium (CAS 1846565-00-1)-containing NO-releasing cream SB414 (6%, NOVAN, Inc, Durham, NC) was performed on the skin for three days (day 2–4), with 4 h between the IMQ and the NO cream applications. Controls were treated with a vehicle cream.

### Statistics

Significance was determined using Student’s t*‐*test or one-way ANOVA followed by Dunnett’s or Šídák’s multiple comparisons test. A *p* value of ≤0.05 was considered significant. The data are presented as the mean ± standard error of the mean (SEM) and statistical analyses were performed using GraphPad Prism v9.1.2 (GraphPad Software, La Jolla, CA).

## Results

### NOS2 mRNA is strongly expressed in the epidermis of psoriasis-involved skin

**Fig. 1:**
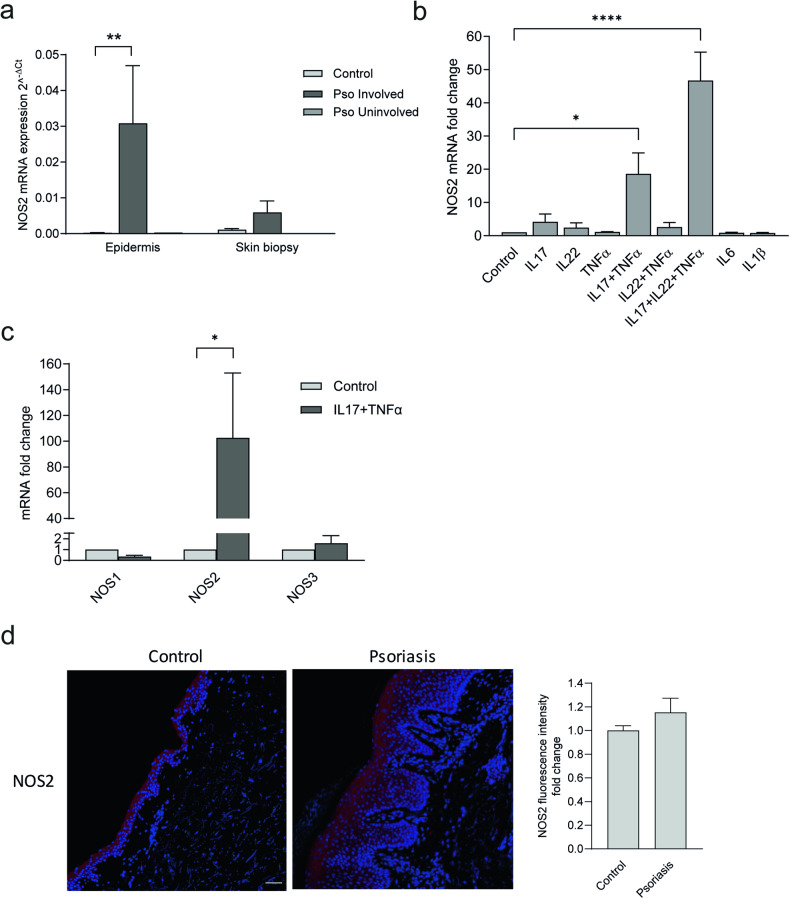
NOS2 protein level does not correlate to the mRNA level in psoriatic skin. **a** *NOS2* mRNA analyses of epidermis and whole-skin biopsies from psoriasis patients (involved and uninvolved tissues) and healthy controls. epidermis *n* = 3, skin biopsies *n* = 4 **b** *NOS2* gene expression in human epidermal keratinocytes (HEKn) treated with the cytokines IL-17, IL-22, TNFα, IL-6 for 48 h. *n* = 3. **c** *NOS1, NOS2* and *NOS3* gene expression in IL-17- and TNFα-treated HEKn. *n* = 3. **d** Immunohistochemical NOS2 staining of psoriatic and control human skin. Nuclei were counterstained with DAPI (blue). bar = 50 µm. For analyzing fluorescence intensity, the epidermis part was defined in the ImageJ software, and the intensity was correlated to this area. controls *n* = 4, patients *n* = 8. Statistical analysis was performed using one-way ANOVA with Šidák’s (**a**, **c**) or Dunnett’s (**b**) multiple comparisons test or Student’s *t*-test (**d**). *p < 0.05, **p < 0.01, ****p < 0.0001.

To delineate the expression of NOS2 in psoriatic skin at the mRNA level, we analyzed the expression in skin biopsies (both in whole-skin and epidermis separately) from psoriatic and healthy control skin, using qPCR. We found a tendency towards NOS2 upregulation in psoriatic whole-skin biopsies, compared with normal whole skin (Fig. 1a). Specifically, a significant, very strong NOS2 expression was detected in involved psoriatic epidermis compared with uninvolved and control epidermis (Fig. 1a), suggesting that the epidermis is the major source of NOS2 in psoriatic skin.

### NOS2 mRNA is induced by IL-17 in keratinocytes

We hypothesized that stimuli present in the psoriatic microenvironment contribute to the strong NOS2 mRNA expression in the psoriatic epidermis. To explore this, human epidermal keratinocytes (HEKn) were stimulated with psoriasis-associated cytokines (IL-17, IL-22, TNFα, IL-6 and IL-1β) for 48 h. We found that IL-17 in combination with TNFα significantly induced *NOS2* mRNA expression almost 20-fold compared with control and that the addition of IL-22 potentiated this induction to more than 45-fold (Fig. 1b). The effect of cytokines was validated by increased expression of *CXCL1* and *S100A8* (Fig. S1). IL-17 was indispensable for the induction, since IL-22, neither alone nor in combination with TNFα, was able to induce NOS2. No change in NOS2 expression was detected by IL-1β or IL-6 stimulation. Next, we examined whether other environmental factors present in the psoriatic micromilieu influence NOS2 expression in keratinocytes. Neither starvation, hypoxia, reactive oxidative species (ROS) nor calcium deficiency influenced the expression level of NOS2 (Fig. S2). Furthermore, we found that IL-17 did not upregulate the expression levels of the other major isoforms of nitric oxide synthase, NOS1 and NOS3 (Fig. 1c), which suggests that NOS2 is the main source of NO in our experimental setting. Our data suggest that IL-17 is a key NOS2-inducing stimulus in psoriatic keratinocytes.

### NOS2 protein level does not correlate to the mRNA level in psoriatic skin

Immunohistochemical analysis was performed on paraffin-embedded sections of psoriasis-involved and control skin. Surprisingly, the NOS2 expression in psoriatic epidermis was only slightly upregulated at the protein level compared to control, without reaching statistical significance (Fig. 1d). The same expression pattern and intensity of NOS2 in the psoriatic epidermis was found using four different antibodies. Due to the volatile nature of NO, we were unable to detect its presence in the epidermis after processing (removal of the epidermis with dispase overnight, data not shown). Next, we stained psoriatic sections with antibodies against nitrotyrosine (3-NT), which is a marker of cellular damage in the presence of high NO levels. We found no significant difference in expression between psoriatic lesions and normal skin (Fig. S3). Our data thus suggest that the upregulated mRNA expression in psoriasis is not fully translated into NOS2 protein.

### miR-31 is induced by IL-17 and reduce NOS2 translation in keratinocytes

We hypothesized that the discrepancy between the high mRNA expression in psoriatic epidermis and the lack of a corresponding increase in protein expression was due to the inhibition of its translation. MicroRNAs (miRNA) are small non-coding RNAs that act as important posttranscriptional gene regulators through the repression of protein translation or by reducing mRNA stability [13]. To test our hypothesis, we selected two of the most abundantly overexpressed miRNA in psoriatic epidermis, miR-31 and miR-146a [14, 15], of which miR-146a was previously implicated in NOS2 regulation in mouse renal cells [16].

**Fig. 2:**
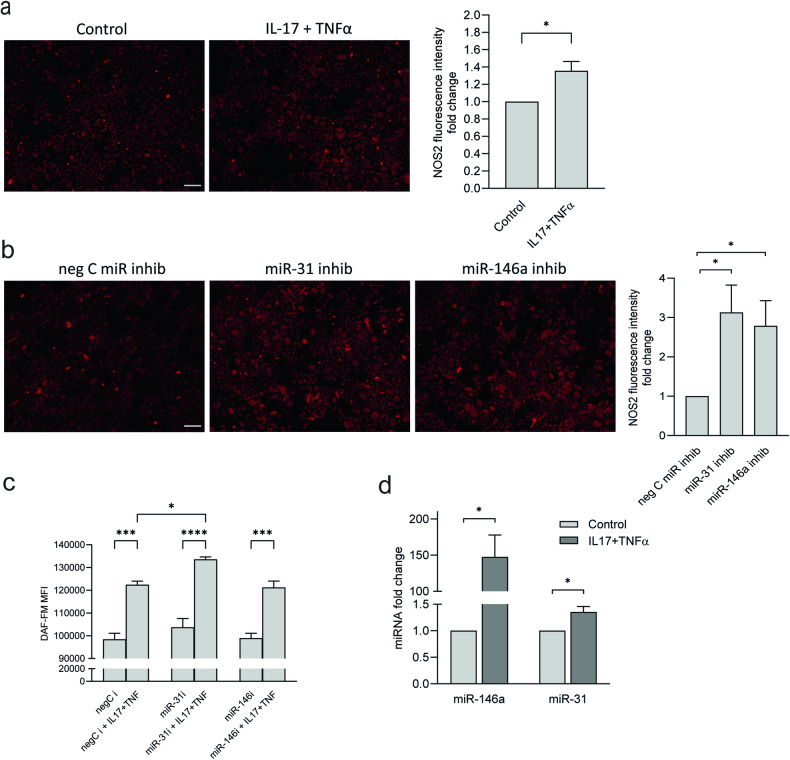
miRNA-31 reduces NOS2 protein expression. NOS2 immunostaining (red) of human keratinocytes (**a**) stimulated with IL-17 + TNFα for 48 h or (**b**) transfected with miR-31, miR-146a or negative control (neg C) miRNA inhibitors before IL-17 + TNFα stimulation. A fluorescence intensity measurement related to the number of cells was performed and compared as the fold change to control. *n* = 3, bar = 150 µm. **c** Nitric oxide (NO) production was analyzed in miRNA inhibitor (miR-146a, miR-31 and negative control) transfected and IL17 + TNFα treated cells and detected by DAF-FM fluorescence using Cytek Aurora flow cytometry. Mean fluorescence intensity (MFI) was measured. *n* = 3. **d** miR-146a and miR-31 expression in response to IL-17 + TNFα. *n* = 4 Statistical analysis was performed using Student’s *t*-test (**a**, **b**, **d**) or one-way ANOVA with Šidák’s multiple comparisons test (**c**). *p < 0.05, **p < 0.01.

HEKn cells were stimulated with the combination of IL-17 and TNFα for 48 h, which led to a modest increase in NOS2 protein expression, using immunocytochemistry and Western blot analyses (Figs. 2a and S4a). By transfecting the cells with mirVana miR-146a and miR-31 inhibitors, which completely silenced the respective miRNA expression (Fig. S5), we found a significant increase in NOS2 protein expression after cytokine stimulation compared with control cells transfected with the negative control miRNA inhibitor (Fig. 2b). Western blot analysis showed increased NOS2 protein levels upon inhibition of miR-31, but without reaching statistical significance (Fig. S4b). Using flow cytometry, we demonstrated a significant induction of NO, as a readout for NOS2, upon treatment with IL-17+TNFa, and a significant further potentiation by miR-31 inhibition (Fig. 2c). Further, we demonstrated that the IL-17 stimulation of HEKn induced miR-31 and miR-146a expression (Fig. 2d). Upon silencing of miR-146a, we observed upregulated NOS2 protein expression using immunofluorescence but could not validate these findings using western blot analysis. In addition, we found no significant NO increase in the miR-146a inhibitor transfected cells, which implies that miR-146a may not play an essential role in the regulation of NOS2 protein expression. Taken together, the immunocytochemistry, Western blot, and flow cytometry analyses suggest that miR-31 prevent the full translation of IL-17-induced NOS2 mRNA expression in HEKn. The effect of miR-31 on NOS2 protein expression is likely to be indirect, since no predicted miR-31 target sites were found in the NOS2 transcript using miRTarBase v9.0.

### Modestly increased NO levels promote keratinocyte proliferation and colony formation

**Fig. 3:**
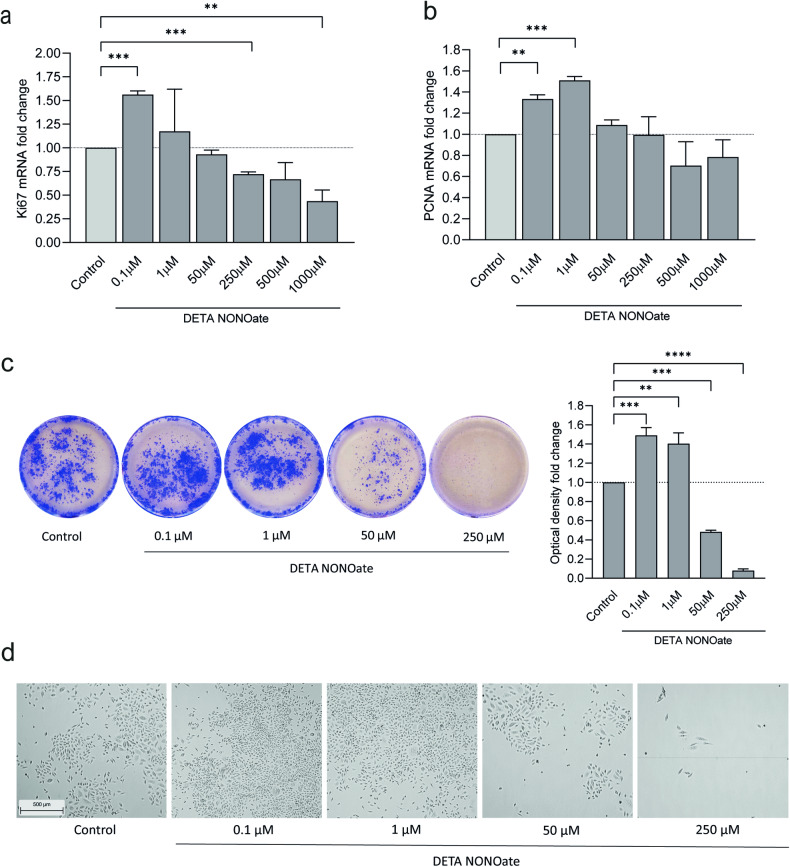
Increased proliferation by low-dose nitric oxide. Gene expression of the proliferation markers **a** *Ki67* *n* = 3 and **b** *PCNA* *n* = 3 in human keratinocytes treated for 48 h with different concentrations of the NO donor DETA-NONOate (0.1-1000 µM) *n* = 3. For proliferation experiments, cells were sparsely seeded and grown for 8 days in the presence of 0.1–1000 µM NO donor. **c** Crystal violet staining and **d** photographs of the cells were performed *n* = 3. The results were validated by dissolving the stain and spectrophotometrically measuring it. bar = 500 µm. Statistical analysis was performed using one-way ANOVA with Dunnett’s multiple comparisons test. ****p < 0.0001, ***p < 0.001, **p < 0.01.

The expected slightly upregulated NO levels in psoriatic skin led us to explore the consequences of different NO levels on the cellular proliferation of keratinocytes, by analyzing the proliferation markers Ki67 and PCNA. We demonstrated that stimulation with the NO donor DETA-NONOate in the range of 0.1–1 µM led to significantly increased Ki67 mRNA expression, whereas levels in the range of 500–1000 µM led to decreased expression (Fig. 3a). We found a similar expression pattern for PCNA (Fig. 3b). These results were confirmed in a colony assay, in which keratinocytes were sparsely seeded and cultured for 8 days in the presence of different concentrations of the NO donor. After staining with crystal violet, we detected significantly more and larger colonies of cells cultured in the presence of 0.1 µM and 1 µM NO and much fewer and smaller colonies when cultured in higher concentrations of NO (500 µM and 1000 µM), compared with controls (Fig. 3c). NO-induced cell proliferation is also visualized in Fig. 3d, which shows dense cell growth using lower NO levels and viable, yet sparse cell growth using higher NO levels.Our data raise the hypothesis that the incomplete translation of NOS2 mRNA through miRNA-mediated repression leads to increased proliferation in psoriatic keratinocytes.

### NOS2-generated NO regulates IL-17 downstream genes in keratinocytes

**Fig. 4:**
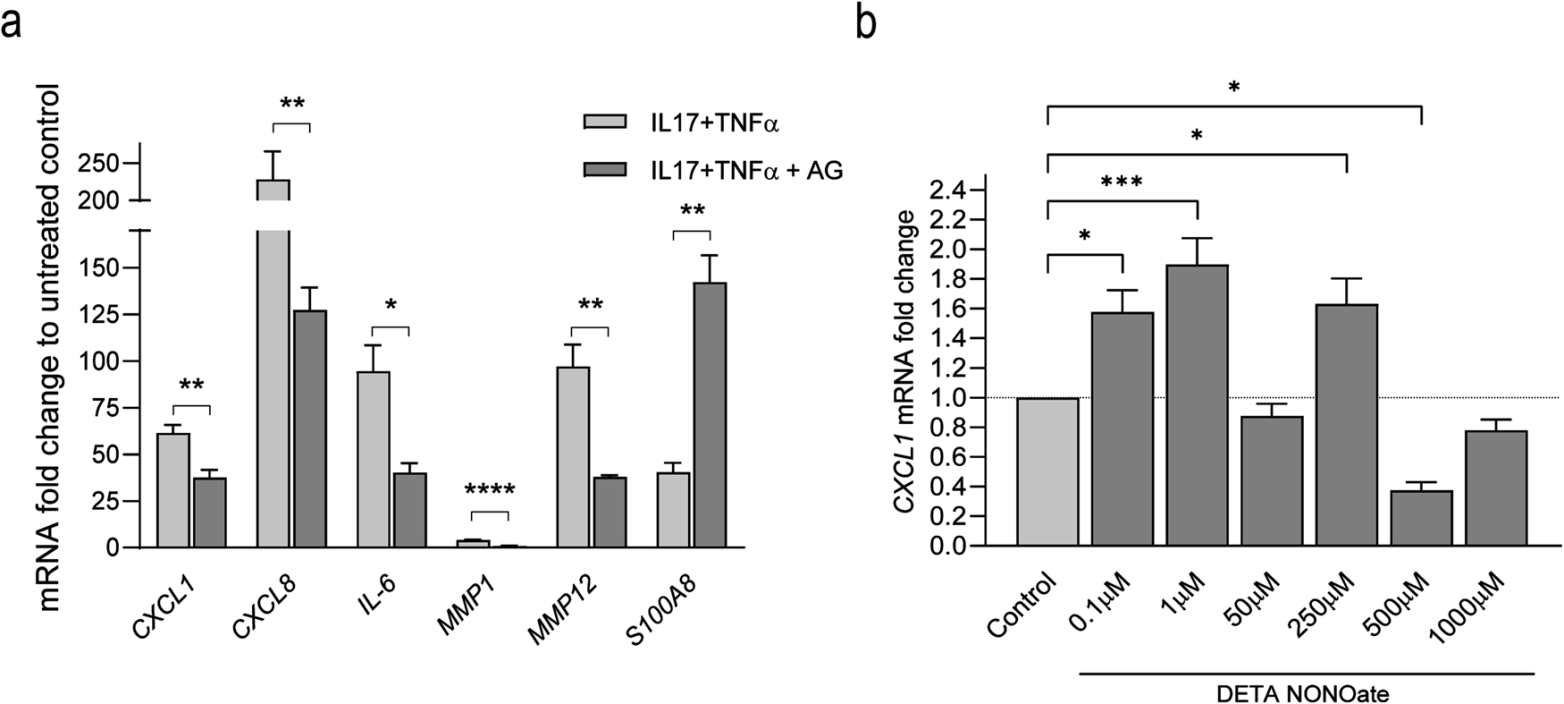
NOS2 regulates IL17-downstream genes. **a** Expression of the IL17-regulated and psoriasis-associated genes *CXCL1, CXCL8, IL-6, MMP1, MMP12 and S100A8* in human keratinocytes treated with the NOS2 inhibitor aminoguanidine (AG) and IL-17 in combination with TNFα. *n* = 3 **b** *CXCL1* expression was analyzed in keratinocytes in response to different concentrations of the NO donor DETA-NONOate (0.1-1000 µM). *n* = 3. Statistical analysis was performed using Student’s *t*-test (**a**) or one-way ANOVA with Dunnett’s multiple comparisons test (**b**). ****p < 0.0001, ***p < 0.001, **p < 0.01, *p < 0.05.

Since NOS2 and NO were induced by IL-17 stimulation, we explored whether NOS2 may mediate IL-17 downstream effects. We used the NOS2 inhibitor aminoguanidine (AG) in IL-17-stimulated HEKn and analyzed genes representing different groups of IL-17 downstream target genes [17]. The analyzed genes (*CXCL1, CXCL5, CXCL8, S100A8, BD2, MMP1, MMP12, IL-6, LCN2*) were selected for also being overexpressed in psoriatic lesions [18]. We found a significant decrease in *CXCL1, CXCL8, IL-6, MMP1 and MMP12* expression and an increase in *S100A8* expression in cells treated with IL-17 in combination with TNFα after NOS2 inhibition (Fig. 4a). The downregulation of *CXCL1* and *MMP1* by NOS2 inhibition was confirmed at the protein level (Fig. S6). To confirm that NOS2-generated NO mediates these effects of IL-17, we treated HEKn with the NO donor DETA-NONOate and found a significant increase in the mRNA expression of *CXCL1* by lower levels (0.1–1 µM) of NO and reduced *CXCL1* by higher levels (500–1000 µM) (Fig. 4b). Our findings suggest that lower levels of NOS2-derived NO mediate the regulation of IL-17 downstream genes, such as *CXCL1* and *MMP1*.

### Alleviated psoriatic phenotype in IMQ-treated NOS2 KO mice and in mice treated with IMQ and an NO-releasing cream

**Fig. 5:**
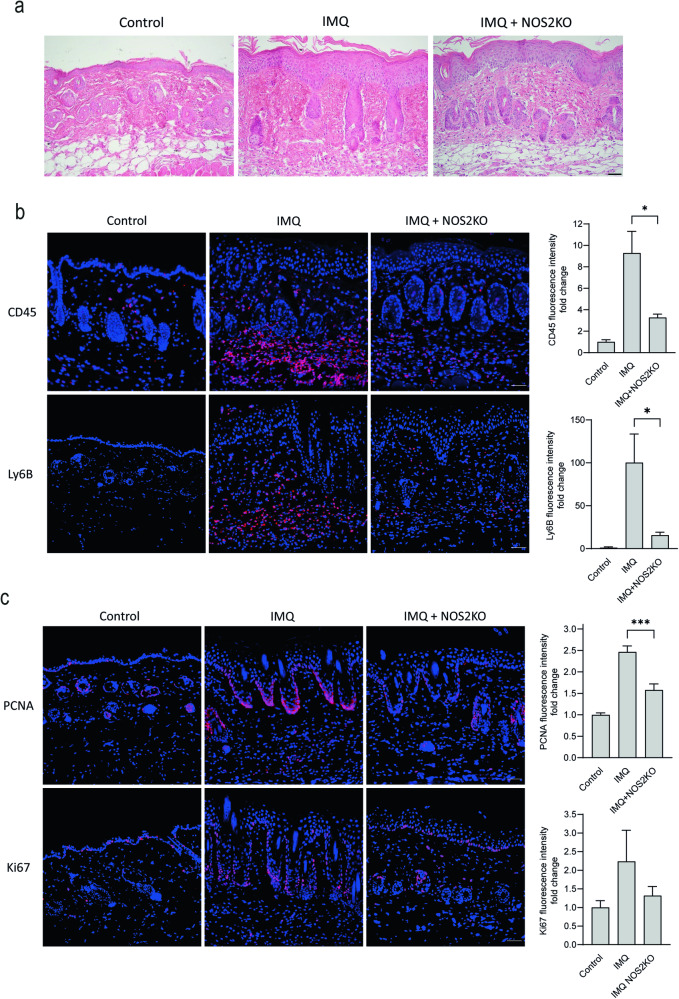
NOS2 is involved in psoriasis-like inflammation and proliferation in mice. The skin of NOS2 knockout (KO) and control mice was treated with imiquimod (IMQ) to induce skin inflammation. **a** Hematoxylin- and eosin-stained skin sections (*n* = 6). The sections were fluorescently stained for (**b**) CD45+ (*n* = 6) and Ly6b+ (*n* = 6) immune cells and (**c**) the proliferation markers PCNA (*n* = 6) and Ki67 (*n* = 4). Nuclei were counterstained with DAPI (blue). Fluorescence intensity measurements were performed and compared as the fold change to control. bar = 50 µm. Statistical analysis was performed using one-way ANOVA with Šidák’s multiple comparisons test. ***p < 0.001, *p < 0.05.

Based on the finding that modestly increased NO levels as opposed to higher levels drive IL-17-mediated effects, we hypothesized that psoriatic inflammation would be alleviated by either eliminating or increasing cellular NO levels in the skin. To test these two conditions in vivo, we used the imiquimod (IMQ) psoriasis mouse model, which generates Th17-driven psoriasiform inflammation [19]. Like in human psoriasis, we found that the NOS2 protein expression was not evidently upregulated in response to IMQ (Fig. S7). To investigate the condition in which NO is absent, we used NOS2 KO mice. In control mice, IMQ induced histological characteristics of psoriatic inflammation, such as increased epidermis thickness and a dense dermal inflammatory cell infiltration with dilated vessels, which was reduced in NOS2 KO mice (Fig. 5a). Immunofluorescence staining showed markedly decreased CD45+ hematopoietic cells, and significantly lower number of neutrophils, indicating that the CD45+ cells primarily consisted of neutrophils (Fig. 5b). Furthermore, in NOS2 KO mice, we found a lower number of both PCNA- and Ki67-positive cells, suggesting reduced epidermal proliferation (Fig. 5c). There were no differences in NOS2 KO control mice compared with wild-type mice. Our data highlight the fact that the limited NOS2 protein expression present in IMQ-treated skin contributes significantly to the psoriatic phenotype.

**Fig. 6:**
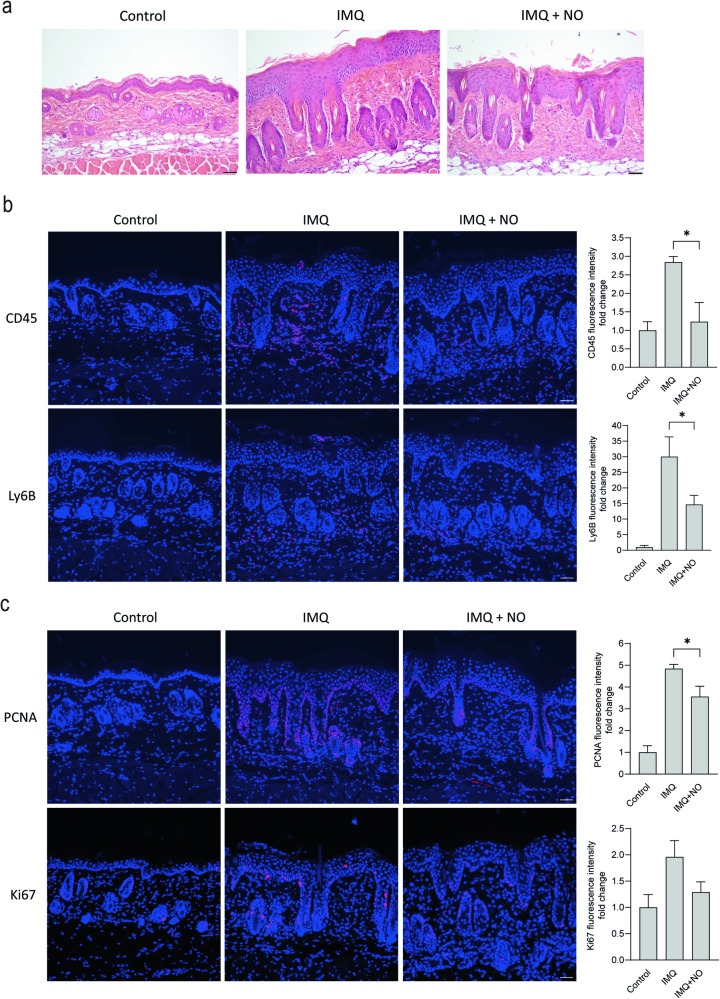
An increased level of NO reduces psoriasis-like inflammation and keratinocyte proliferation in mice. **a** Hematoxylin- and eosin-stained skin sections (*n* = 6). An NO-releasing cream, berdazimer, was applied on imiquimod (IMQ)-treated mouse skin. The sections were stained for (**b**) CD45+ (*n* = 6) and Ly6b+ (*n* = 6) immune cells and (**c**) the proliferation markers PCNA (*n* = 6) and Ki67 (*n* = 6). Nuclei were counterstained with DAPI (blue). Fluorescence intensity measurements were performed and compared as the fold change to control. bar = 50 µm. Statistical analysis was performed using one-way ANOVA with Šidák’s multiple comparisons test. *p < 0.05.

To investigate the effects of increased cellular NO levels in psoriatic inflammation in vivo, we used the NO-releasing berdazimer cream, SB414 (Novan, Inc). In this experiment, IMQ was applied first to establish psoriasis-like skin lesions. Then, starting on the second day of IMQ administration, IMQ and the berdazimer cream were added in parallel until mice were sacrificed at day 5. The effects of IMQ in the positive control were dampened by the vehicle control cream, which served as a moisturizer. As in the NOS2KO mice, we found no significant difference in skin thickness in NO donor cream-treated IMQ mice compared with IMQ-only-treated mice (data not shown). However, the mice treated with the NO-releasing cream displayed signs of normalization of keratinocyte differentiation, with partially decreased parakeratosis and restored granular layer (Fig. 6a), and a reduced dermal density of collagen bundles and inflammatory infiltrate. Moreover, we detected significantly fewer CD45+ cells and neutrophil infiltrate (Fig. 6b), as well as reduced keratinocyte proliferation, as observed as significantly lower PCNA protein expression (Fig. 6c). No effects were observed when applying the NO-donor cream on control skin. Furthermore, the berdazimer cream significantly increased the 3-NT staining in IMQ-treated skin, supporting the presence of enhanced NO levels in the skin (Fig. S8). As a result, both the NOS2 KO and NO donor cream experiments suggest that either eradicating or enhancing the cellular NO level reduces the psoriasis-like inflammation and proliferation in the skin.

## Discussion

**Fig. 7:**
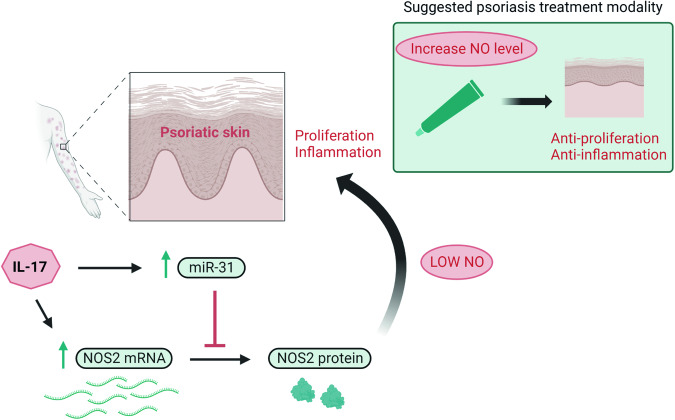
Schematic figure illustrating the proposed mechanism for the regulation of NOS2 expression by IL-17 and the subsequent release of low concentration of NO that may contribute to the pathogenesis of psoriasis. The figure also includes the suggested psoriasis treatment modality using a NO-releasing cream. This figure was created with BioRender.com.

Psoriasis is characterized by keratinocyte hyperproliferation, a process driven by IL-17 and IL-22 [20, 21]. We previously described an overall more immature phenotype of proliferating psoriatic keratinocytes and demonstrated how IL-17 and IL-22 act on keratinocytes to promote proliferation and keep the cells in an immature state [22]. Here, we report that IL-17 induces the expression of the psoriasis susceptibility gene *NOS2* in keratinocytes and that NOS2-derived NO mediates IL-17 downstream effects (Fig. 7). IL-17 was previously shown to induce NO and NOS2 mRNA in mouse and rat endothelial cells [23] and in human chondrocytes [24].

Despite the high NOS2 mRNA expression in psoriatic epidermis, we found no corresponding NOS2 protein expression and no indirect signs of high NOS2 activity, in terms of a high nitrotyrosine level. We demonstrated that the psoriasis-associated miRNA, miR-31, was induced by IL-17 and prevented the full translation of the IL-17-induced NOS2 mRNA.

Our data imply that NOS2-derived NO is present at marginally increased levels in psoriatic skin. We found that stimulation with a lower level of NO (0.1–1 µM DETA-NONOate), as opposed to a higher level (500–1000 µM DETA-NONOate), increased the proliferation of keratinocytes and colony formation, suggesting that a modestly increased NO concentration contributes to the psoriatic phenotype. In line with these data, we demonstrated that a lower level of NO, in contrast to higher levels, mediated IL-17 downstream psoriatic effects. IL-17 thus induces NOS2 and fine-tunes its translation towards a window of proinflammatory and hyperproliferative effects.

Based on this data, we hypothesized that psoriatic inflammation would be alleviated by moving away from the proinflammatory window, by either totally removing cellular NO or increasing it to an anti-inflammatory concentration range. Using the IMQ psoriasis mouse model in NOS2 KO mice, we found a reduced neutrophilic infiltrate and a lower expression of epidermal Ki67 and PCNA, suggesting that the NOS2 protein expression, which was present at low levels in response to IMQ in control mice, contributes to the psoriatic phenotype.

Next, we increased cellular NO levels in the skin by the topical application of an NO-releasing cream (Novan, Inc). Although only applied three times on established psoriasis-like skin, we found a similar effect on the psoriatic inflammation and proliferation as in the NOS2 KO mice, supporting the hypothesis that a continuous application of an NO-releasing cream would alleviate psoriatic inflammation.

In line with our data, using a mannan-induced psoriasis mouse model (MIP), Zhong *et al*. showed that inhibition of NOS2 led to a suppression of skin inflammation [25]. The mechanism of the MIP model is associated with activated macrophages, whereas the IMQ model we used is dependent on Rorγt- ILCs and γδ T cells [26]. In contrast to their study, we demonstrated therapeutic effects of both inhibited and increased NO levels on the psoriatic epidermal inflammation in mice and found NO-mediated effects on IL-17 downstream genes in keratinocytes for modestly increased NO levels. Based on our data, we suggest a novel therapeutic approach to increase NO levels in keratinocytes that are accessible by topical application.

There has been an assumption that NOS2 is highly expressed in psoriatic skin. This assumption is based on the well-documented overexpression of NOS2 on the mRNA level compared with uninvolved skin areas and with healthy control skin [27–29]. However, reports regarding the protein expression in keratinocytes using immunohistochemistry are limited. While some studies only describe localization, others describe increased NOS2 protein staining focally in psoriatic skin [27, 30]. We used four different antibodies, of which two have previously been used on skin [6, 31], to confirm our findings. The lack of strong overexpression is also supported by two recent proteomic analyses of psoriatic skin and epidermis [32, 33]. In addition to the lack of confirmation of NOS2 overproduction at the protein level, the consequence of the expected high level of NO because of NOS2 protein overproduction has likewise not been verified in previous studies [32, 33]. For instance, citrulline, an end product of NO synthesis from NOS2, was found to be equally present in psoriatic skin and in control skin [34]. On the other hand, NOS2 has been convincingly shown to be expressed by CD11c+ dendritic cells present in both epidermis and dermis (CD11c+/TNFa+ Tip-DCs) [7, 35, 36].

NO has been identified as a regulator of cell proliferation and differentiation in many cell types [37–39]. Moreover, a biphasic effect of NO on proliferation has been previously observed [39–43]. Thus, using the NO donor DETA-NONOate in a micromolar range favors proliferation, whereas using it in a millimolar range mediates antiproliferative and even toxic effects. In contrast, physiological concentrations of NO are estimated to be below 50 nM [38]. In keratinocytes, low NO was suggested to induce proliferation but not fibroblasts [40]. The growth-inhibiting activity in keratinocytes by NO at higher concentrations was suggested by showing the reduction of Ki67 in organ cultures of normal human skin in the presence of 1000 µM DETA-NONOate [27].

While much progress has been made in the development of novel biological treatments for patients with severe disease, there are few topical treatment options for the majority of patients with mild-to-moderate psoriasis. Moreover, a substantial number of patients need topical treatment options based on comorbidities that preclude systemic therapy.

There is increased interest in the development of topical NO donor therapies for treating a broad spectrum of skin diseases, including atopic dermatitis and wound healing, in which NO promotes the proliferation of dermal fibroblasts and endothelial cells and thus promotes angiogenesis [44, 45]. The nitric oxide-releasing berdazimer gel, SB206 (Novan, Inc) was previously evaluated in a phase 2 study of molluscum contagiosum in 250 children and in a phase 3 study including 891 patients, with both low and mild adverse events and favorable efficacies [46, 47]. We evaluated the NO-releasing cream, SB414 (Novan, Inc), containing berdazimer sodium, the same active ingredient as SB206, in the IMQ mouse model and found a reduction in the psoriatic phenotype after three daily applications.

In conclusion, we demonstrate the proof of concept of NO donor therapy in a preclinical psoriasis mouse model and provide a mechanism for the proliferation-dampening effects of elevated NO levels. These results pave the way towards a new topical treatment modality that could benefit the majority of patients with mild-to-moderate psoriasis.

### Supplementary information

Supplemental figures and legends

Original data WB

## Data Availability

All data generated or analyzed during this study are included in this published article and its supplementary material.
