# Supplementary material for: NOS2-derived low levels of NO drive psoriasis pathogenesis
Source: Cell Death Dis. 2024 Jun 26;15(6):449. doi: 10.1038/s41419-024-06842-z (PMC11208585; doi:10.1038/s41419-024-06842-z)
Supplement: Supplementary file 1 — Supplemental figures and legends [file 41419_2024_6842_MOESM1_ESM.pdf]

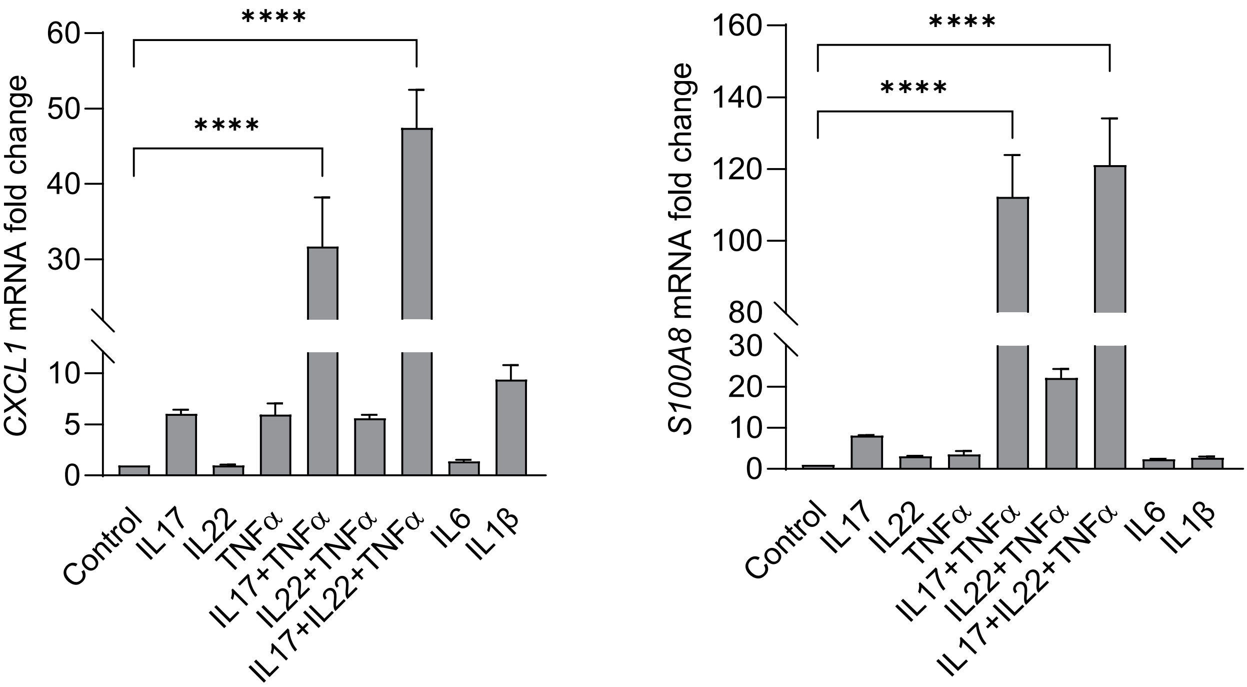

CXCL1 mRNA fold change
60
50
40
30
10
5
0
****
****
Control
IL17
IL22
TNFα
IL17+TNFα
IL22+TNFα
IL17+IL22+TNFα
IL6
IL1β
S100A8 mRNA fold change
160
140
120
100
80
30
20
10
0
****
****
Control
IL17
IL22
TNFα
IL17+TNFα
IL22+TNFα
IL17+IL22+TNFα
IL6
IL1β

Figure S1

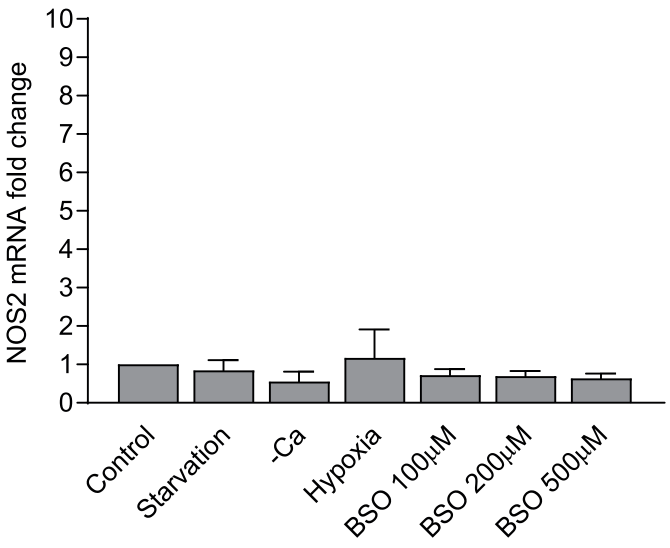
NOS2 mRNA fold change
10
9
8
7
6
5
4
3
2
1
0
Control
Starvation
-Ca
Hypoxia
BSO 100μM
BSO 200μM
BSO 500μM

Figure S2

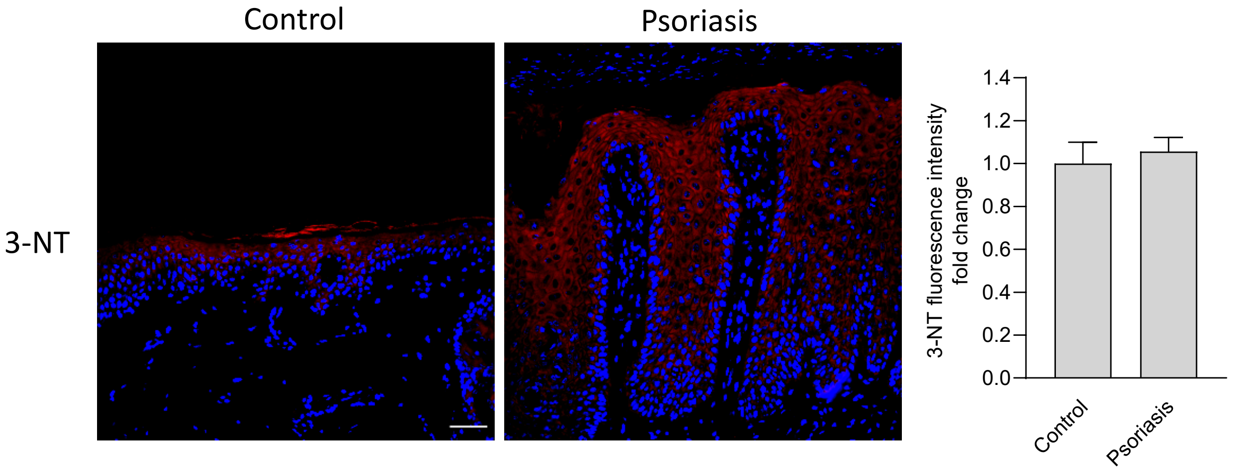

Control
Psoriasis
3-NT
3-NT fluorescence intensity fold change
1.4
1.2
1.0
0.8
0.6
0.4
0.2
0.0
Control
Psoriasis

Figure S3

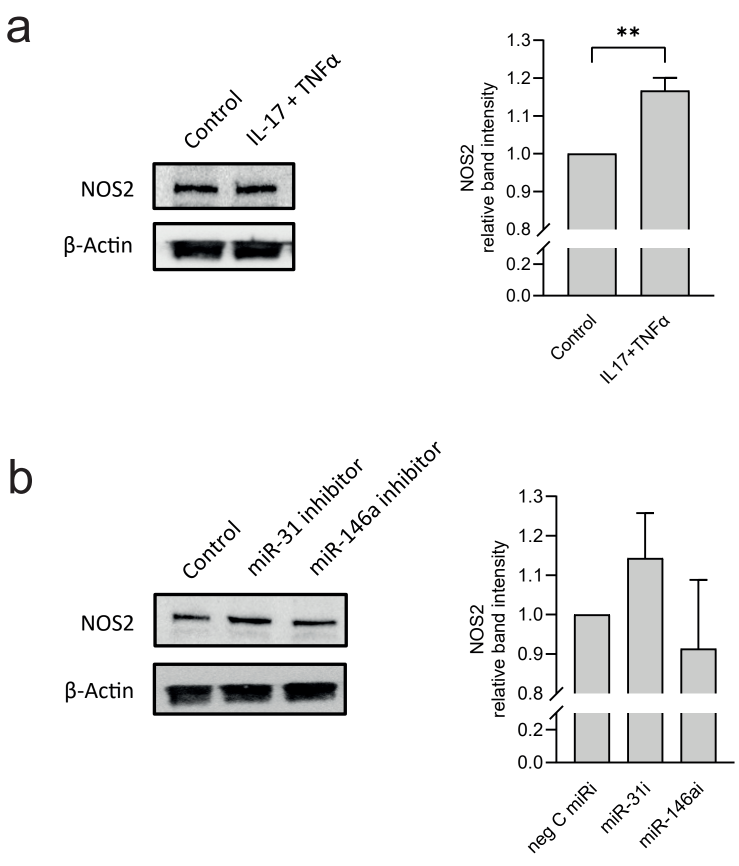
a
Control
IL-17 + TNFα
NOS2
β-Actin
**
1.3
1.2
1.1
1.0
0.9
0.8
0.2
0.0
NOS2
relative band intensity
Control
IL17+TNFα
b
Control
miR-31 inhibitor
miR-146a inhibitor
NOS2
β-Actin
1.3
1.2
1.1
1.0
0.9
0.8
0.2
0.0
NOS2
relative band intensity
neg C miRi
miR-31i
miR-146ai

Figure S4

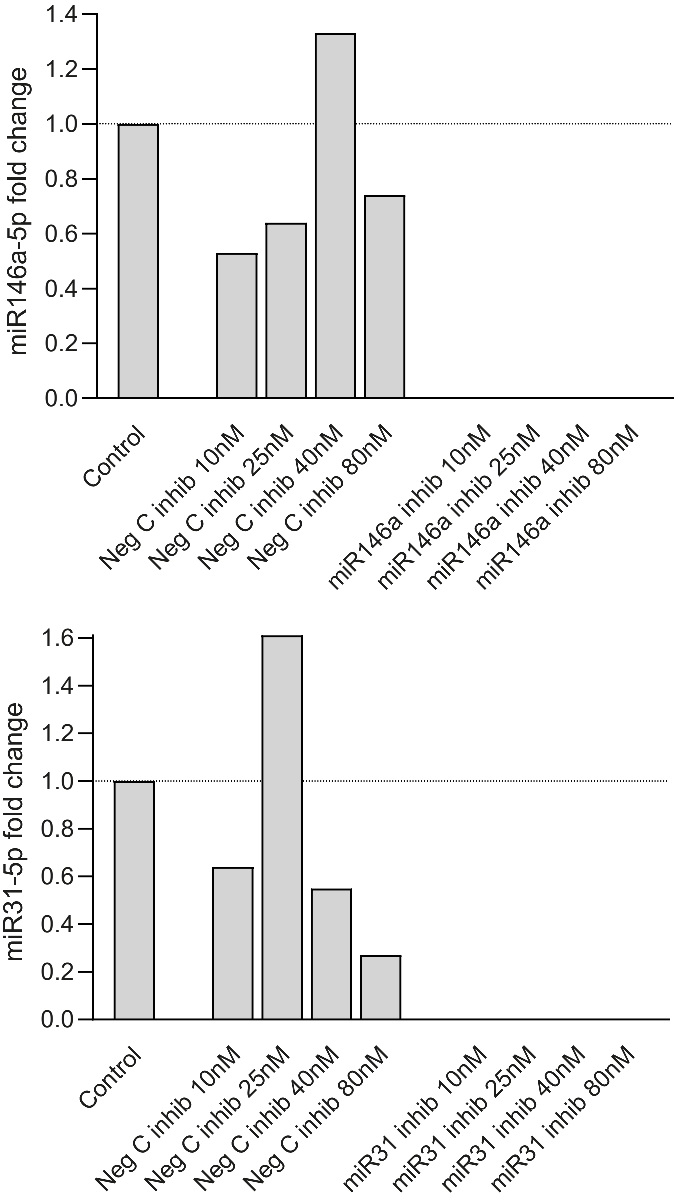

miR146a-5p fold change
1.4
1.2
1.0
0.8
0.6
0.4
0.2
0.0
Control
Neg C inhib 10nM
Neg C inhib 25nM
Neg C inhib 40nM
Neg C inhib 80nM
miR146a inhib 10nM
miR146a inhib 25nM
miR146a inhib 40nM
miR146a inhib 80nM
miR31-5p fold change
1.6
1.4
1.2
1.0
0.8
0.6
0.4
0.2
0.0
Control
Neg C inhib 10nM
Neg C inhib 25nM
Neg C inhib 40nM
Neg C inhib 80nM
miR31 inhib 10nM
miR31 inhib 25nM
miR31 inhib 40nM
miR31 inhib 80nM

Figure S5

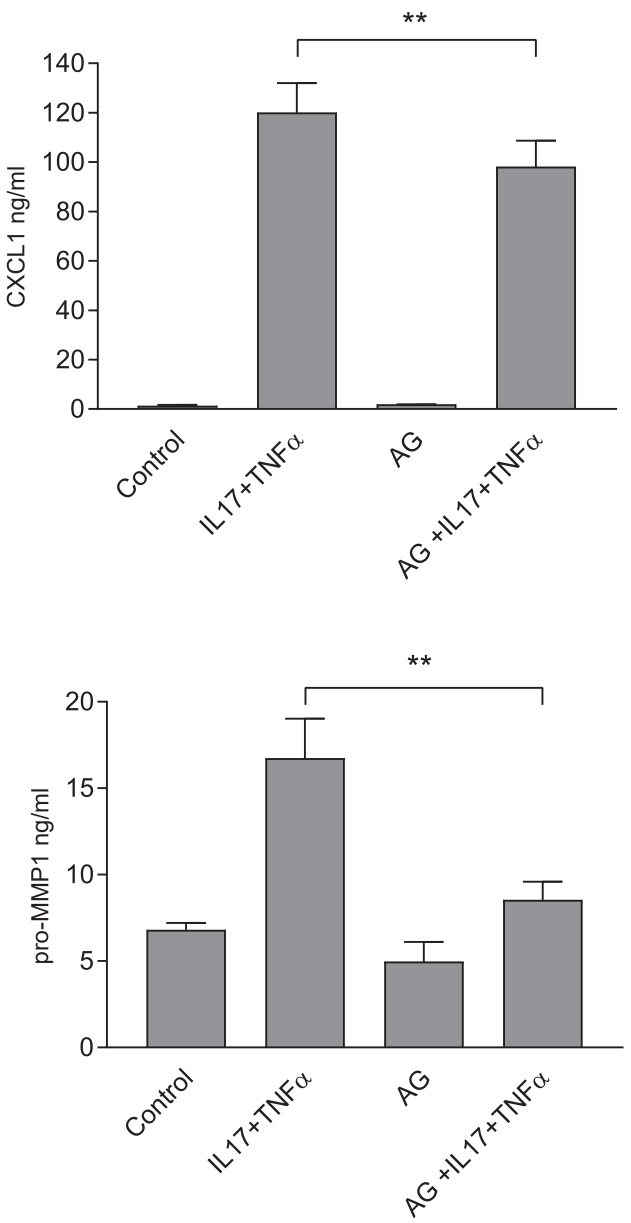
**
140
120
100
80
60
40
20
0
CXCL1 ng/ml
Control
IL17+TNFα
AG
AG +IL17+TNFα
**
20
15
10
5
0
pro-MMP1 ng/ml
Control
IL17+TNFα
AG
AG +IL17+TNFα

Figure S6

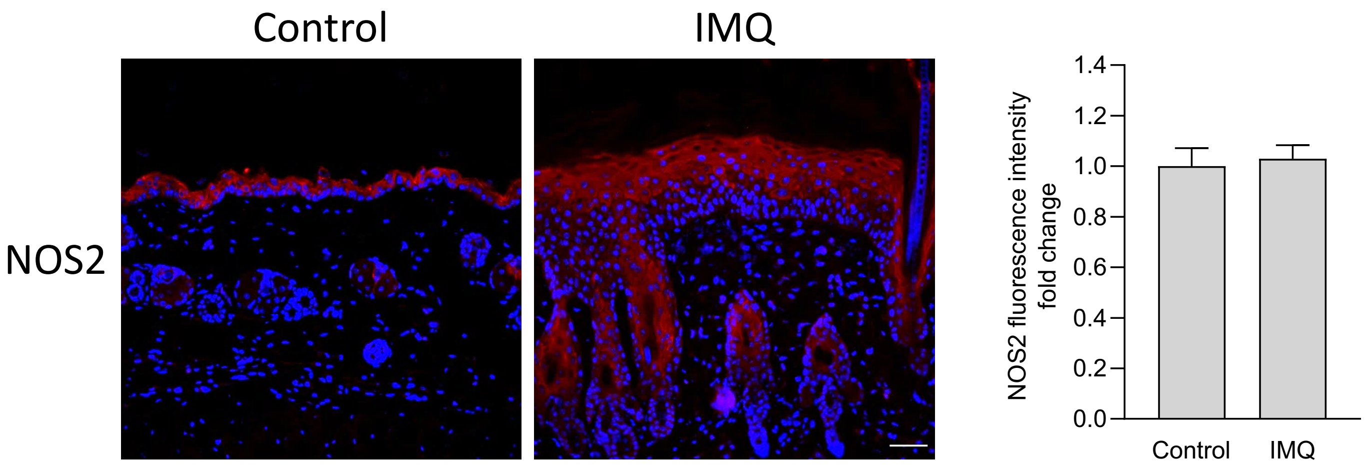

Control
IMQ
NOS2
NOS2 fluorescence intensity fold change
1.4
1.2
1.0
0.8
0.6
0.4
0.2
0.0
Control
IMQ

Figure S7

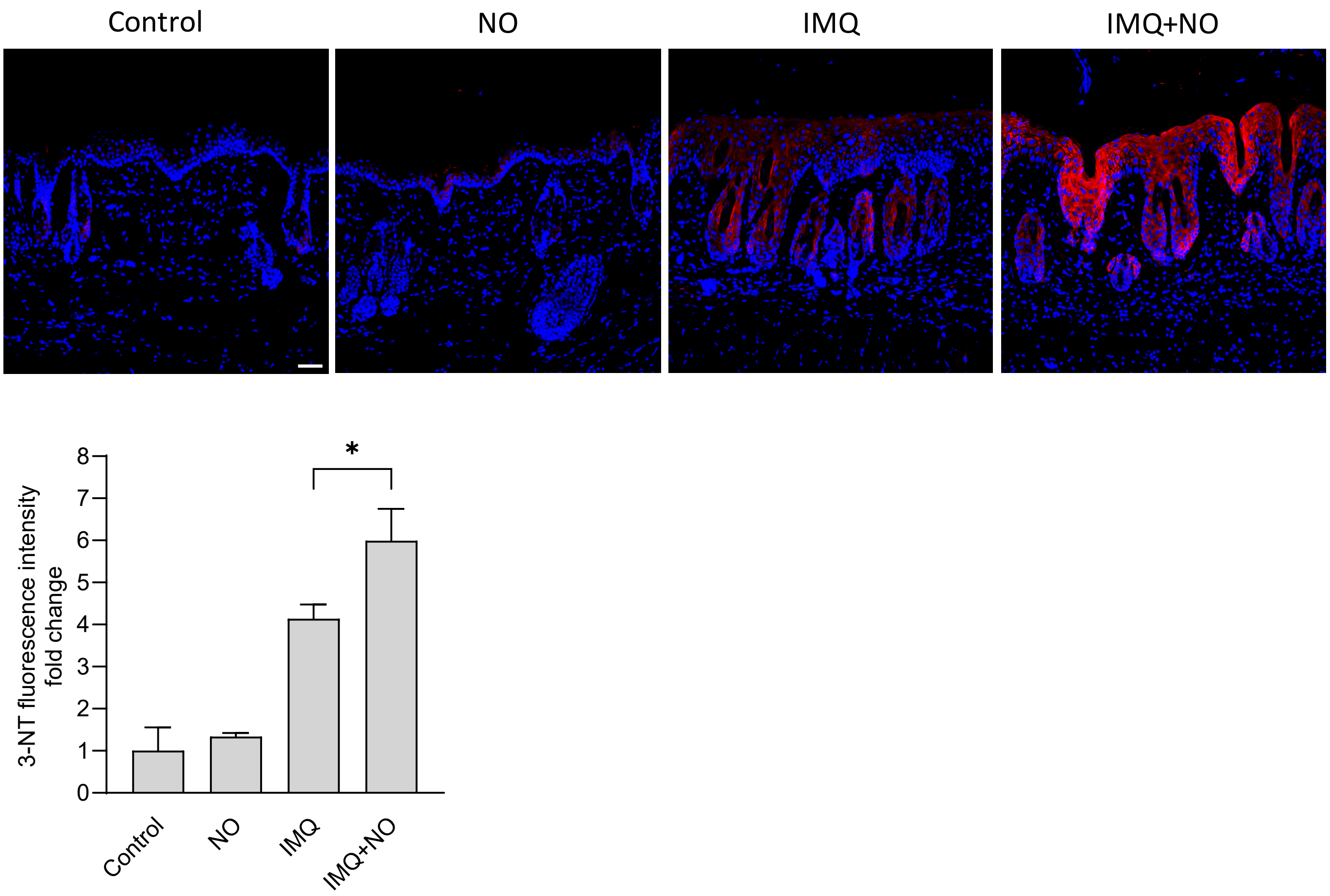
Control
NO
IMQ
IMQ+NO
*
3-NT fluorescence intensity
fold change
8
7
6
5
4
3
2
1
0
Control
NO
IMQ
IMQ+NO

Figure S8

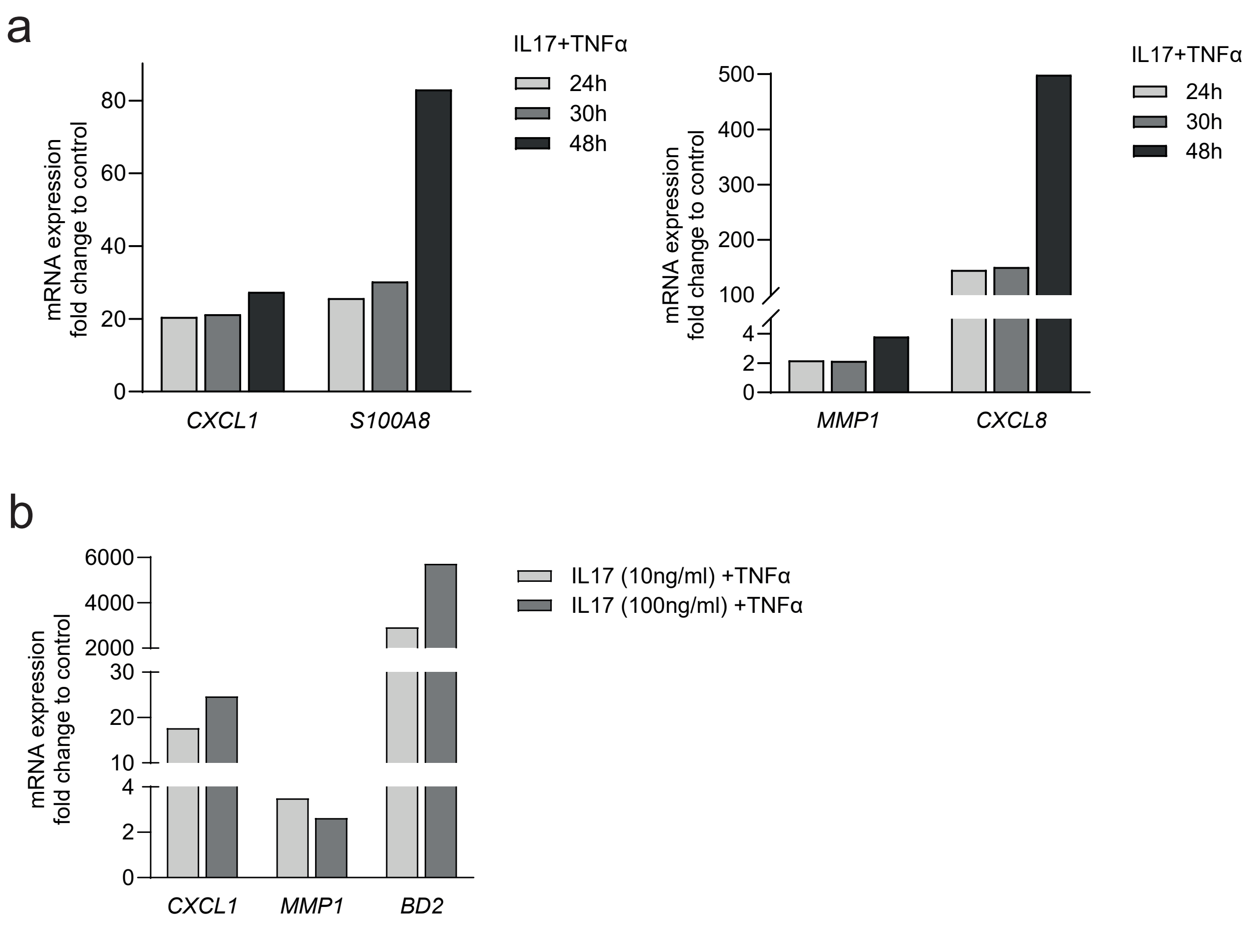
a
IL17+TNFα
24h
30h
48h
mRNA expression
fold change to control
0
20
40
60
80
CXCL1
S100A8
IL17+TNFα
24h
30h
48h
mRNA expression
fold change to control
0
2
4
100
200
300
400
500
MMP1
CXCL8
b
IL17 (10ng/ml) +TNFα
IL17 (100ng/ml) +TNFα
mRNA expression
fold change to control
0
2
4
10
20
30
2000
4000
6000
CXCL1
MMP1
BD2

Figure S9

FIGURE LEGENDS TO SUPPLEMENTAL FIGURES

Figure S1. *CXCL1* and *S100A8* gene expression in human epidermal keratinocytes (HEKn) in response to different cytokines. Statistical analysis was performed using one-way ANOVA with Dunnett's multiple comparisons test. n=3, ****p<0.0001

Figure S2. *NOS2* gene expression in human epidermal keratinocytes (HEKn) treated with different environmental factors for 48 h. Statistical analysis was performed using one-way ANOVA with Dunnett's multiple comparisons test. n=3

Figure S3. No detectable 3-NT increase in psoriatic skin. Immunohistochemical nitrotyrosine (3-NT) staining of psoriatic and control human skin. Nuclei were counterstained with DAPI (blue). bar = 50 µm. For analyzing fluorescence intensity, the epidermis part was defined in the ImageJ software, and the intensity was correlated to this area. Statistical analysis was performed using Student's t-test. n=3

Figure S4. NOS2 protein expression in response to (**a**) IL-17 and TNFα and (**b**) the miRNA inhibitors miR-31 and miR-146a, determined by Western blot analysis. Band intensities were quantified by densitometry using Image Lab Software. β-actin was used as internal control. Statistical analysis was performed using Student's t-test (a) or one-way ANOVA with Šidák's multiple comparisons test. n=3, *p<0.01

Figure S5. The optimization of silencing miR-146a and miR-31 by miRNA inhibitor transfection. The expression of miR-146a and miR-31 in human keratinocytes transfected with miR-146a and miR-31 inhibitors respectively at different concentrations and treated with IL-17 and TNFα.

Figure S6. ELISA analyses of CXCL1 and MMP1 protein expression in cell supernatants from human keratinocytes treated with the NOS2 inhibitor aminoguanidine (AG) and IL-17 in combination with TNFα. Statistical analysis was performed using Student's t-test. n=3, **$p<0.01$

Figure S7. NOS2 expression in the imiquimod (IMQ)-treated mouse skin. Nuclei were counterstained with DAPI (blue). A fluorescence intensity measurement was performed and compared as the fold change to control. Statistical analysis was performed using Student's t-test. n=3, bar = 50 μm

Figure S8. Increased NO level in the epidermis by topical application of the NO-releasing cream berdazimer on mouse skin. Berdazimer, cream was applied on imiquimod (IMQ)-treated mouse skin and the sections were stained for 3-NT and nuclei were counterstained with DAPI (blue). bar = 50 μm. For analyzing fluorescence intensity, the epidermis part was defined in the ImageJ software, and the intensity was correlated to this area. Statistical analysis was performed using one-way ANOVA with Šidák's multiple comparisons test. *$p<0.05$

Figure S9. Time course (**a**) and dose response (**b**) studies of IL-17 and TNFα stimulation. The expression of the IL-17-induced genes *CXCL1*, *S100A8*, *MMP1* and *CXCL8* were analyzed.
