# Supplementary figures and images for: NOS2-derived low levels of NO drive psoriasis pathogenesis
Source: Cell Death Dis. 2024 Jun 26;15(6):449. doi: 10.1038/s41419-024-06842-z (PMC11208585; doi:10.1038/s41419-024-06842-z)

Uncropped Western blot, Figure S4

NOS2

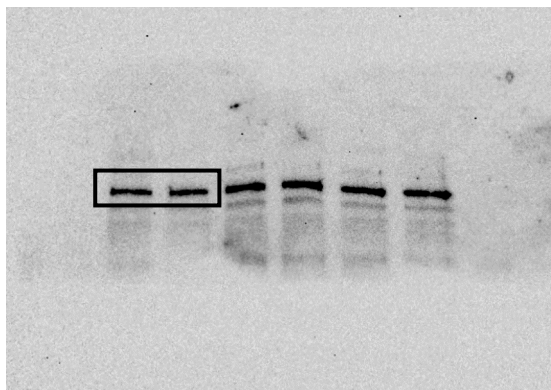

$\beta$ -Actin

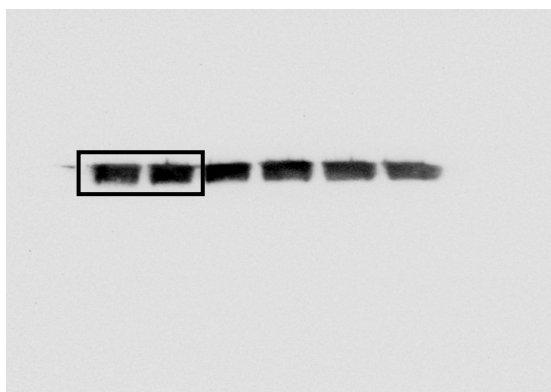

Uncropped Western blot, Figure S4

NOS2

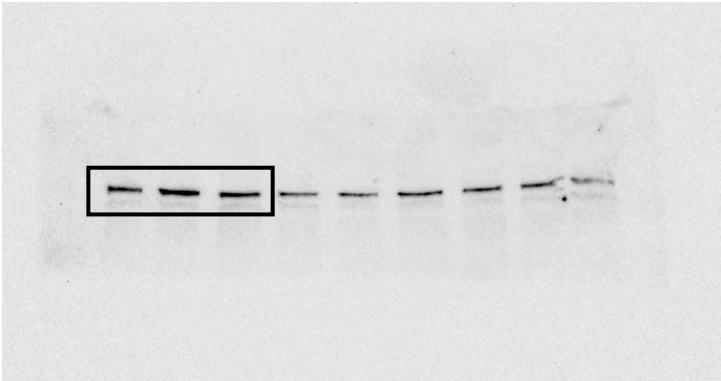

$\beta$ -Actin

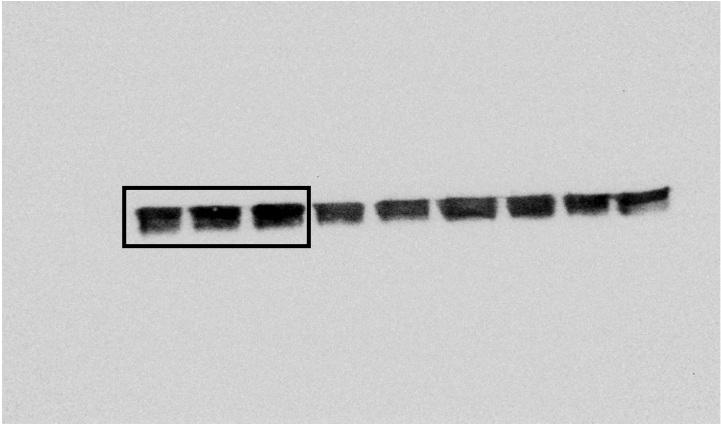

Supplement: Supplementary file 2 — Original data WB [file 41419_2024_6842_MOESM2_ESM.pdf]
